# YbfA Regulates the Sensitivity of *Escherichia coli* K12 to Plantaricin BM-1 *via* the BasS/BasR Two-Component Regulatory System

**DOI:** 10.3389/fmicb.2021.659198

**Published:** 2021-08-17

**Authors:** Xinyue Chen, Yifei Liu, Junhua Jin, Hui Liu, Yanling Hao, Hongxing Zhang, Yuanhong Xie

**Affiliations:** ^1^Beijing Laboratory of Food Quality and Safety, Beijing Key Laboratory of Agricultural Product Detection and Control of Spoilage Organisms and Pesticide Residue, College of Food Science and Engineering, Beijing University of Agriculture, Beijing, China; ^2^Beijing Advanced Innovation Center for Food Nutrition and Human Health, College of Food Science and Nutritional Engineering, China Agricultural University, Beijing, China

**Keywords:** bacteriocins, proteome, YbfA, BasS/BasR two-component, biofilm

## Abstract

Plantaricin BM-1, a class IIa bacteriocin produced by *Lactobacillus plantarum* BM-1, shows obvious antibacterial activity against *Escherichia coli*. However, the mechanism underlying the action of class IIa bacteriocins against gram-negative bacteria remains to be explored. The purpose of this study was to investigate the role of YbfA, a DUF2517 domain-containing protein, in the response of *Escherichia coli* K12 to plantaricin BM-1. The growth curve experiment and MIC experiment showed that the sensitivity of *E. coli* to plantaricin BM-1 was decreased by a *ybfA* null mutation. Electron microscopy showed that the *ybfA* null mutation reduced the surface rupture and contraction caused by plantaricin BM-1, and mitigated the effect of plantaricin BM-1 on the morphology of the *E. coli* cell membrane. Proteomics analysis showed that 323 proteins were differentially expressed in *E. coli* lacking the *ybfA* gene (*P* < 0.05); 118 proteins were downregulated, and 205 proteins were upregulated. The metabolic pathways containing the upregulated proteins mainly included outer membrane proteins, integral components of the plasma membrane, regulation of cell motility, and regulation of locomotion. The metabolic pathways involving the downregulated proteins mainly included outer membrane protein glycine betaine transport, amino-acid betaine transport, and transmembrane signaling receptor activity. The results of the proteomics analysis showed that the protein expression of the BasS/BasR two-component system was significantly increased (*P* < 0.05). Moreover, the expression levels of downstream proteins regulated by this two-component system were also significantly increased, including DgkA, FliC, and MlaE, which are involved in cell membrane structure and function, and RT-qPCR also confirmed this result. The growth curve showed that the sensitivity of *E. coli* to plantaricin BM-1 was significantly increased due to deletion of the BasS/BasR two-component system. Thus, deletion of *ybfA* in *E. coli* can increase the expression of the BasS/BasR two-component system and positively regulate the structure and function of the cell membrane to reduce the sensitivity to plantaricin BM-1. This will help to explore the mechanism of action of class IIa bacteriocins against gram-negative bacteria.

## Introduction

Bacteriocins are secreted hydrophobic antimicrobial peptides, are 20–60 amino acids in length, and are synthesized by bacterial ribosomes. These peptides have been shown to inhibit both gram-negative and gram-positive food pathogens (Tagg et al., 1995). Bacteriocins produced by lactic acid bacteria (LAB) are regarded as potential natural preservatives because of their high bacteriostasis and low toxicity (Tagg et al., 1995). These bacteriocins can not only be used in food processing and storage but also are expected to be an alternative to antibiotics due to their antiseptic and antibacterial properties (Cotter et al., 2013; Liu et al., 2017).

Bacteriocins can be divided into classes I–IV according to their chemical structure, molecular mass, and stability (Klaenhammer, 1993). Class II bacteriocins are unmodified peptides that are resistant to high temperatures, with or without a leading strand (except for disulfide bond modifications). Class IIa bacteriocins are small molecular weight, thermally stable peptides with strong antibacterial activity against *Listeria* (Klaenhammer, 1993), and class IIa bacteriocins from LAB are the most abundant and extensively studied bacteriocins. Mature class IIa bacteriocins contain 37–48 amino acids, and the peptide chain is roughly divided into two regions: a positively charged, highly conserved hydrophilic N-terminus and a poorly conserved amphiphilic or hydrophobic C-terminus (Nes et al., 2007). In the highly conserved N-terminal region, there is a “streptococcin box,” which has a YGNGV/L consensus sequence, and two cysteines form a disulfide bond (Nes et al., 2007). At present, there are two model mechanism for the action of class IIa bacteriocins against gram-positive bacteria; the first one is the “barrel-stave” model, in which the hydrophobic part of the bacteriocin interacts with the hydrophobic core of the cell membrane, resulting in the formation of holes in the surface of the cell membrane (Liu et al., 2013a,b), and the second is the “carpet” model, in which the bacteriocin isn't inserted into the hydrophobic core of the plasma membrane, causing the cell membrane to disintegrate without hole formation (Zhang et al., 2012). However, the mechanism of class IIa bacteriocins in gram-negative bacteria remains to be explored.

Two-component systems (TCSs) are one of the most common mechanisms by which bacteria perceive, react, and adapt to environmental changes (Mizuno, 1997). A typical TCSs is composed of a sensor kinase (histidine kinase, HK) located on the inner membrane and a cytoplasmic response regulator (RR) (Mizuno, 1997; Beier and Gross, 2006; Merighi et al., 2009; Gotoh et al., 2010). In most systems, the HK perceives environmental stimuli and autophosphorylates on a conserved histidine residue. Then, the phosphoryl group is transferred to the conserved aspartic acid residue on its homologous RR (Gotoh et al., 2010). The BasS/BasR two-component system is a typical TCS that functions as an iron-zinc induced transcription regulator which directly regulates a group of genes related to metal responsive membrane structure modification and membrane function regulation in *E. coli* K12 (Yu, 2019). In addition, the BasS/BasR two-component system can also upregulate the expression of genes related to biofilm formation in *E. coli* (Yu, 2019).

*Lactobacillus plantarum* BM-1 was isolated from a traditional fermented meat product. It produces a new type IIa bacteriocin, plantaricin BM-1, which has significant inhibitory activity against some foodborne bacteria, including *E. coli* (Zhang et al., 2013). In our previous study, we found that the expression of YbfA, a protein containing the DUF2517 domain and expressed by a 207 bp gene *ybfA*, was obviously upregulated in *E. coli* treated with plantaricin BM-1 (Wang et al., 2020a,b). In this study, we mainly investigated the regulatory role of YbfA in sensitivity to plantaricin BM-1 in *E. coli*. According to the results, YbfA upregulated the sensitivity of *E. coli* K12 to plantaricin BM-1 *via* the BasS/BasR two-component regulatory system.

## Materials and Methods

### Reagents

The chemical and biochemical reagents used in the experiment are shown in Table 1. The chemicals and reagents used were all analytically pure.

**Table 1:** Main experimental reagents and sources.

**Primary reagents**	**Factory**
Antibiotics	TransGen Biotech CO., Ltd
Plasmid extraction kit	TIANGEN BIOTECH (BEIJING) CO., Ltd
Bacterial genomic DNA extraction kit	TIANGEN BIOTECH (BEIJING) CO., Ltd
TIANgel Midi Purification Kit	TIANGEN BIOTECH (BEIJING) CO., Ltd
DNA Marker	Takara Biomedical Technology (Beijing) Co., Ltd
TaKaRa Ex Taq	Takara Biomedical Technology (Beijing) Co., Ltd
Bacteria RNA Extraction Kit	Vazyme Biotech CO., Ltd
HiScript II One Step qRT-PCR SYBR Green Kit	Vazyme Biotech CO., Ltd

### Strains and Cultivation Conditions

The strains and plasmids used in this study are listed in Table 2. *Escherichia coli* K12 and *E. coli* JW0688 were cultured in Luria-Bertani (LB) broth at 37°C with aeration at 180 rpm. *Lactobacillus plantarum* BM-1 was cultured in de Man, Rogosa, and Sharpe (MRS) broth at 37°C with aeration at 180 rpm.

**Table 2:** Strains and plasmids used in this study.

**Strains and plasmids**	**Characteristics**	**Source**
*E. coli* K12	Wild-type *E. coli* strain BW25113	Laboratory preservation
*E. coli* JW0688	*E. coli* BW25113 with *ybfA* deletion	Keio collection Tomoya et al., 2006
*E. coli* JW4073	*E. coli* BW25113 with *basS* deletion	Keio collection Tomoya et al., 2006
*E. coli* JW4074	*E. coli* BW25113 with *basR* deletion	Keio collection Tomoya et al., 2006
*L. plantarum* BM-1	*L. plantarum* BM-1, produces plantaricin BM-1	Laboratory preservation
pKD46	Plasmid containing the lambda Red system, L-arabinose inducible	BioVector NTCC
*E. coli* ReJW0688	*E. coli* JW0688 with *ybfA* complemented	This study

### Determination of Minimal Inhibitory Concentration (MIC)

The minimum inhibitory concentration (MIC) was determined according to Clinical and Laboratory Standards Institute (CLSI) guidelines (CLSI, 2012). Plantaricin BM-1 was purified using a two-step method. According to our previous study (Zhang et al., 2013), the method of preparing plantaricin BM-1 is briefly described as follows: *L. plantarum* BM-1 was cultured in sterile MRS broth at 37°C for 12 h, centrifuged at 10,000 r/min at 4°C for 10 min, and the culture medium was collected. The plantaricin BM-1 was purified through pH-mediated cell adsorption–desorption and cation- exchange chromatography on an SP Sepharose Fast Flow column. The purified plantaricin BM-1 was freeze-dried and stored at −80°C.

The concentration of purified plantaricin BM-1 was quantified using a NanoDrop ND-1000 spectrophotometer. Then, doubling dilution (39.04, 19.52, 9.74, 4.88, 2.44, 1.22, 0.61, and 0.305 mg/mL) were prepared with sterile water under sterile conditions. Adding diluents to 1–8 columns (100 μL per well) of 96-well plates in a diluted gradient, and the same amount of sterile water was added to column 9 as a blank control. *Escherichia coli* K12 grown to logarithmic growth phase was collected and used to prepare a bacterial suspension at a concentration of 10^4^ CFU/mL. Then, an aliquot of the *E. coli* K12 suspension (100 μL) was added to each well. After mixing, the 96-well plates were incubated at 37°C for 12 h, and the optical density (OD) at 600 nm (OD_600_) was determined using an ELISA plate reader. The minimum concentration of plantaricin BM-1 to *E. coli* K12 and *E. coli* JW0688 at which no bacterial growth was detected (i.e., no increase in the OD_600_) was recorded as the MIC (Lv et al., 2014). Each experiment was repeated three times.

### Construction of a ybfA-Complemented Mutant of *E. coli* JW0688

The *ybfA* complemented mutant in *E. coli JW0688* was constructed using the lambda Red homologous recombination method (Juhas and Ajioka, 2016). Briefly, the pKD46 plasmid was transformed into competent *E. coli* JW0688 prepared with cold 0.1 mol/mL CaCl_2_, and expression of the homologous recombinase in pKD46 was induced by adding L-arabinose (0.5 mg/mL in LB broth) at 30°C. Competent cells of *E. coli* JW0688 containing the pKD46 plasmid were prepared. The *ybfA* gene fragment was amplified from *E. coli* K12 using the primers *ybfA*-F (5′-3′): AAGGGGGAGAAAAGTATGGAACTCTACAGA and *ybfA*-R (5′-3′): AAGTTTTGAGTCGTTTCAATAAAAATCACCA (homology underlined), and then transfected into the prepared *E. coli* JW0688 competent cells. Next, 450 μL of LB broth was added, and the cells were incubated at 37°C for 2 h. Then, the cells were diluted with physiological saline, spread on LB agar, and incubated at 37°C for 12 h. A single colony was selected, and the recombinant was verified using the *ybfA*-F/R primers. The bacterial that containing *ybfA* gene was complemented successfully. And the verified complemented *E. coli* strain was named ReJW0688.

### Bacterial Growth Studies

Plantaricin BM-1 was purified as described by (Zhang et al., 2013). Wild-type *E. coli* K12, *E. coli* JW0688, and *E. coli* ReJW0688, at an initial concentration of 2.0 log_10_ CFU/mL, were cultured in LB broth with or without plantaricin BM-1 (3 × MIC) at 37°C for 14 h. The OD_600_ was measured every 2 h, and the mean values of triplicate experiments were plotted. All experiments were repeated three times.

### Electron Microscopic (EM) Analysis

*Escherichia coli* K12 and *E. coli* JW0688 strains (2.0 log_10_ CFU/mL) were cultured in LB broth with or without plantaricin BM-1 (3 × MIC) at 37°C for 4 h. The bacterial cells were collected by centrifugation (8,000 rpm, 10 min, 4°C), washed three times with 0.1 mol/L PBS, and then prepared for EM analysis (Wang et al., 2020a,b).

*Scanning EM (SEM):* The washed cells were fixed with 2.5% glutaraldehyde at 25°C for 2 h and then washed with PBS (0.1 mol/L) three times. The cells were dehydrated with a graded series of ethanol (50, 70, 80, 90, and 100%, three times). The dehydrated samples were treated with 100% tert-butyl alcohol three times and dried for 2 h in a freeze dryer. The sample was placed on the sample table and coated with a 10 nm gold film using an ion sputter coater (Cao et al., 2018). The specimens were imaged using an SEM (SU8010; Hitachi, Japan).

*Transmission EM (TEM)*: Cells were washed with PBS (0.1 mol/L) three times, fixed with 2.5% glutaraldehyde for 2.5 h, and washed with PBS (0.1 mol/L) three times. The dehydration steps were the same as those described for SEM. After dehydration, the sample was washed with acetone three times, soaked in a mixture of acetone and embedding agent (3:1) for 2 h, and then placed on an embedding plate containing pure embedding agent. The embedding plates were polymerized at 40 and 60°C for 48 h. The sample was processed to a trapezoid shape with a surface area of less than 0.2 × 0.2 mm. The embedded material was ultrathin sectioned to a thickness of 50–90 nm. Finally, the ultra-thin sections were stained with uranium and lead dyes and washed for 5 min (Yi et al., 2018). Imaging and analysis of samples were performed using a TEM (7800; Hitachi, Japan).

### Proteomic Analysis

*Escherichia coli* K12 and *E. coli* JW0688 (2.0 log10 CFU/mL) were cultured in LB broth at 37°C for 12 h. After centrifugation, the collected cells were resuspended in lysate buffer (8 M urea, 1% SDS, protease inhibitor). The samples were ground three times and then dissolved on ice for 30 min. Finally, the treated samples were centrifuged at 10,000 rpm for 30 min at 4°C to isolate the total protein. The total protein concentration was measured using the Pierce bicinchoninic acid (BCA) assay (Thermo Fisher, USA).

A 10 μL protein sample was mixed with 90 μL of lysate buffer and then treated with 10 mM TECP (Thermo Scientific, USA) and 40 mM iodoacetamide (Thermo Scientific, USA) at room temperature for 40 min. The sample was mixed with chilled acetone at a volume ratio of 6:1 (sample:acetone), and incubated at −20°C for 4 h to precipitate protein. The samples were centrifuged at 10,000 rpm for 20 min. The precipitated proteins were completely dissolved in 100 μL of 100 mm TEAB and digested with trypsin at a ratio of 1:50. The peptides (100 μg) were mixed with an appropriate amount of TMT reagent for labeling (Thermo Fisher, USA) and incubated at room temperature for 2 h. Following this, hydroxylamine was added, and the sample was incubated at room temperature for 15 min, and then lyophilized in a vacuum freeze dryer for liquid chromatography with tandem mass spectrometry (LC-MS/MS) analysis. The analysis was conducted using a reversed-phase liquid chromatography system (Thermo Scientific Vanquish Flex; Thermo Scientific, USA) equipped with a reverse phase C18 column (ACQUITY UPLC BEH C18 Column, 1.7 μm, 2.1 × 150 mm; Waters, USA) and high pH liquid phase separation TMT labeling peptide. Peptide elution was monitored at 214 nm, and after 5 min, the eluted peptide fractions were collected once per min. A total of 10 fractions were pooled and lyophilized. The dried fractions were identified and quantified on a Q Exactive MS (Thermo Scientific, USA) using a C18 column (75 μm × 25 cm; Thermo Scientific, USA). The parameters for identification were as previously published (Wu et al., 2018).

The data were processed using Proteome Discoverer^TM^ Software 2.2 (Thermo Fisher Scientific, USA). A protein search was conducted using the UniProt-*E. coli* (strain K12) [83333]-4353s-20190412 database, with a false discovery rate (FDR) ≤0.01. Proteins with at least one unique peptide were used for protein quantification. Differential proteins with fold changes >1.2 (upregulated) or <0.83 (downregulated) were selected. A *P* value less than 0.05 was considered significant. Gene ontology (GO) and KEGG pathway analysis of the proteomics results were performed using BLAST2GO and KOBAS.

### RT-qPCR

Wild-type *E. coli* K12 and mutant *E. coli* JW0688 at an initial concentration of 2.0 log_10_ CFU/mL, were cultured in LB broth at 37°C for 14 h. Total bacterial RNA was extracted for RT-qPCR. The RT-qPCR reaction conditions were 50°C for 15 min, 95°C for 30 s, 95°C for 10 s, 60°C for 30 s, 40 cycles, using primers as shown in Table 3. Using wild-type *E. coli* K12 as internal reference factor, the relative gene expression in mutant *E. coli* JW0688 was calculated by 2^−ΔΔCT^ method.

**Table 3:** Primers for RT-qPCR.

**Primers name**	**Primer sequence (5′-3′)**	**Gene function**
*16s*F	ACCCTTATCCTTTGTTGCC	Reference genes
*16s*R	TCTTTGTATGCGCCATTGTA	
*bass*F *bass*R	ACCCTGCTGCGGATGTTATTGC	Translating sensor protein BasS
	AATACCTGGTCCTTCATCTTCAACTGC	
*basr*F	TTGTTGAAGACGATACGCTGTTATTGC	Translating transcriptional regulatory protein BasR
*basr*R	AAATCCAGTACCACCAGGCTGTAATG	
*dgka*F	GGTATTGTTGGCGGTGGTC	Diacylglycerol kinase Diacylglycerol kinase Diacylglycerol kinase
*dgka*R	TTCGATGGCGCTATTGAGG	
*mlae*F	CGGGTTCTTCTGGTCGGCAATG	Translating intermembrane phospholipid transport system permease protein MlaE
*mlae*R	ACACCACGCTCTTAATCAGACAGTTG	

### Analysis of the Sensitivity of *E. coli* K12 BasS/BasR Two-Component System Mutant to Plantaricin BM-1

The MIC of *E. coli* JW4073 (*basS* null mutant) and *E. coli* JW4074 (*basR* null mutant) to plantaricin BM-1were determinded by using the same method in Determination of minimal inhibitory concentration (MIC).

Three *E. coli* strains, *E. coli* JW4073 (*basS* null mutant), *E. coli* JW4074 (*basR* null mutant), and *E. coli* K12 (at an initial concentration of 2.0 log10 CFU/mL) were treated with 3 × MIC plantaricin BM-1 and cultured in LB broth at 37°C for 24 h. The OD_600_ was measured every 2 h, and the average of the three growth curves was plotted. All experiments were repeated three times.

## Results

### Determination of the Minimal Inhibitory Concentration

After culturing of the bacterial strains with various concentrations of plantaricin BM-1 at 37°C for 12 h, the OD_600_ of each bacterial suspension was determined, and the concentration at which the OD did not increase, indicating that the bacteria were not growing, was recorded as the MIC. The MIC of plantaricin BM-1 in *E. coli* K12 and *E. coli* JW0688 were 1.22 and 8.54 mg/mL respectively.

### Construction and Confirmation of the ybfA Complemented Strains in *E. coli* JW0688

**Figure 1:**
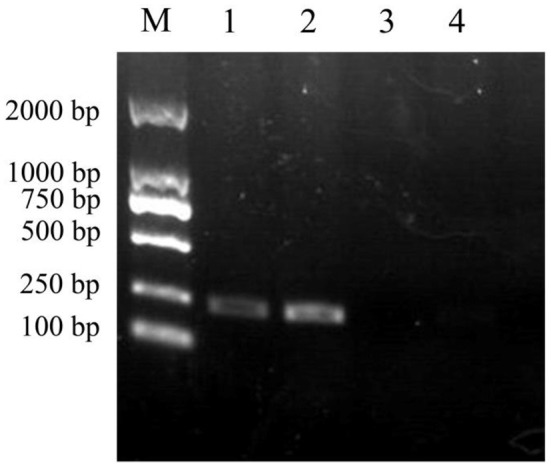
Verification of *E. coli* ReJW0688 construction. PCR products were detected using 2% agarose electrophoresis. Lane M is the 2000 bp DNA Marker, Lanes 1 and 2 contain the PCR product from *E. coli* ReJW0688, and lanes 3 and 4 contain the PCR products from mutant *E. coli* JW0688.

The 207 bp *ybfA* gene fragment was amplified from the *E. coli* K12 genome by PCR using the primer pair *ybfA*-F/R, which was then used to replace the kanamycin resistance gene in *E. coli* JW0688 *via* Red homologous recombination. Genomic DNA was extracted from *E. coli* JW0688 and the complemented strain *E. coli* ReJW0688, and PCR was performed on the two extracted genomes using primers *ybfA*-F/R to confirm proper construction. A 207 bp product was amplified from the complemented strain *E. coli* ReJW0688, but no amplified band was detected in *E. coli* JW0688 (Figure 1). Sequencing showed that the amplified gene fragment from *E. coli* ReJW0688 had the same sequence as the *ybfA* gene fragment in *E. coli* K12, which proved that *E. coli* ReJW0688 was successfully constructed.

### Inhibitory Effect of Plantaricin BM-1 on *E. coli* K12

**Figure 2:**
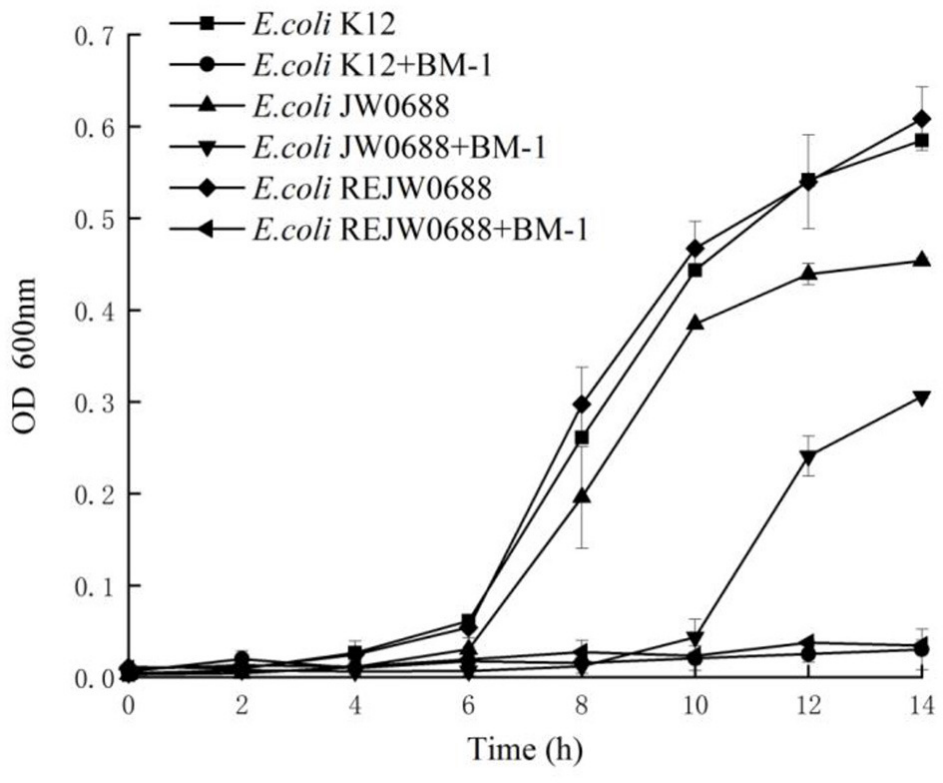
Effect of plantaricin BM-1 on the growth of wild type *E. coli* K12, mutant *E. coli* JW0688, and *E. coli* ReJW0688. ■, ▴ and ♦ represent the OD_600_ values of wild type *E. coli* K12, mutant *E. coli* JW0688, and *E. coli* ReJW0688 in the absence of bacteriocin, respectively. •, ▴ and ◂ represent the OD_600_ values of wild type *E. coli* K12, mutant *E. coli* JW0688, and *E. coli* ReJW0688 in the presence of plantaricin BM-1 (3 × MIC), respectively.

The effects of plantaricin BM-1 on the growth of *E. coli* K12, *E.coli* JW0688, and *E. coli* ReJW0688 were assessed by generating standard growth curves (Figure 2). In the absence of plantaricin BM-1, all three strains showed similar exponential growth. The OD_600_ values of *E. coli* K12, *E. coli* JW0688, and *E. coli* ReJW0688 at 14 h were 0.585, 0.454, and 0.608, respectively. At this time, the growth of *E. coli* JW0688 was slightly lower than that of wild-type *E. coli K12*, which might be caused by some changes in growth metabolism and carbon and nitrogen source absorption after *ybfA* gene mutation was removed. In the presence of plantaricin BM-1, wild-type *E. coli* K12 grew slowly over the 14 h period, and the OD_600_ at 14 h was 0.030, indicating that wild-type *E. coli* K12 is sensitive to plantaricin BM-1. In comparison, *E. coli* JW0688 grew slowly from 0 to 6 h, and then grew rapidly after 6 h, reaching exponential phase. The OD_600_ at 14 h was 0.306, which was significantly (*P* < 0.05) higher than that of wild-type *E. coli* K12. Our results indicated that the *ybfA* null mutation reduced the sensitivity of *E. coli* K12 to plantaricin BM-1. In addition, the growth curve of the complemented strain (*E. coli* ReJW0688) in the presence of plantaricin BM-1 was similar to that of wild-type *E. coli* K12. The growth of *E. coli* ReJW0688 was slow, and the OD_600_ at 14 h was 0.035, which indicated that the sensitivity of the complemented strain was increased to wild-type levels, further indicating that YbfA regulates the sensitivity of *E. coli* to plantaricin BM-1.

### Effect of Plantaricin BM-1 on *E. coli* K12 Morphology

**Figure 3:**
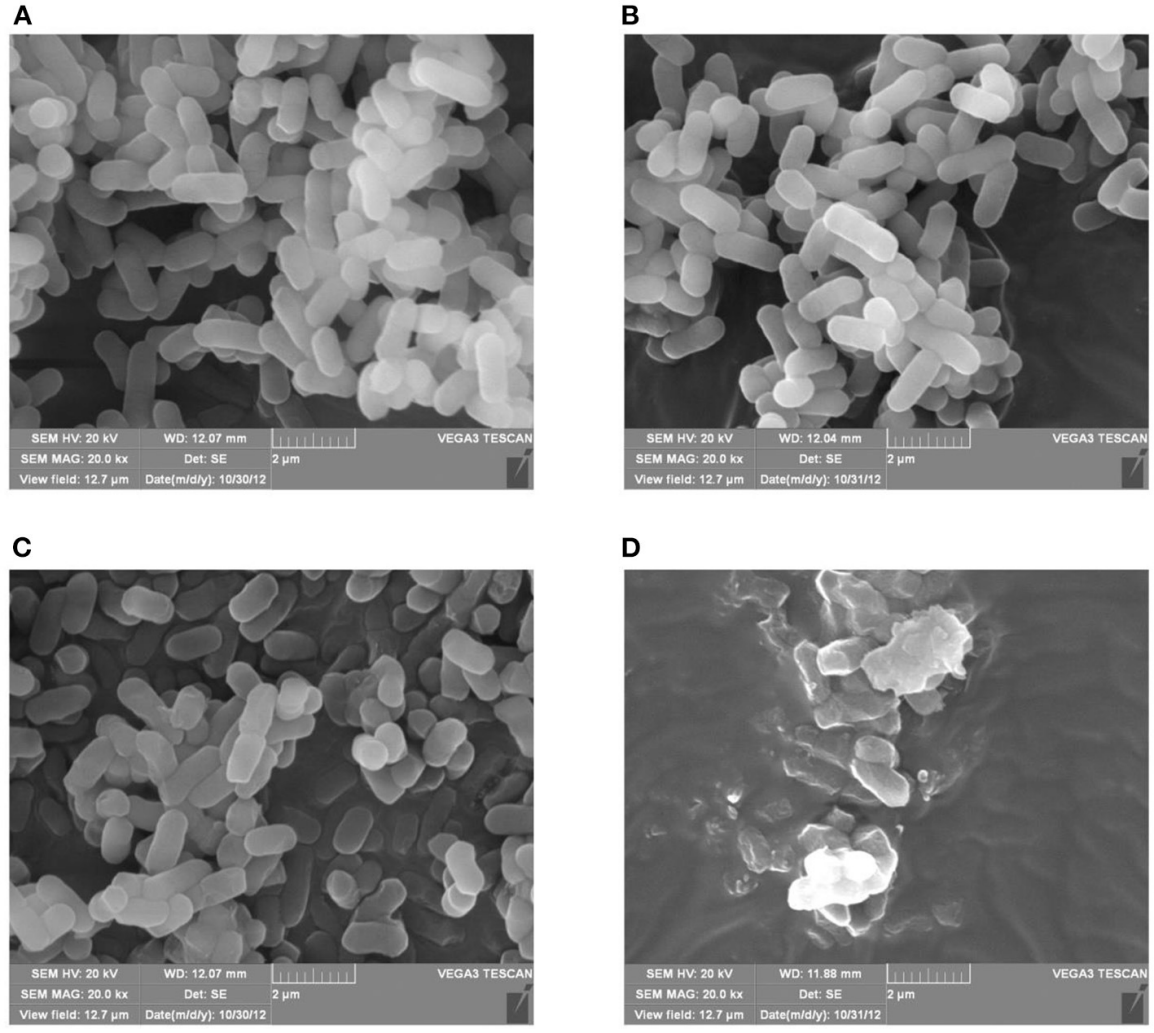
SEM of *E. coli* K12 and *E. coli* JW0688 with and without plantaricin BM-1. **(A,B)** show wild type *E. coli* K12 and *E. coli* JW0688 without plantaricin BM-1, respectively; **(C,D)** show wild type *E. coli* JW0688 and *E. coli* K12 with plantaricin BM-1(magnification: 20,000×).

**Figure 4:**
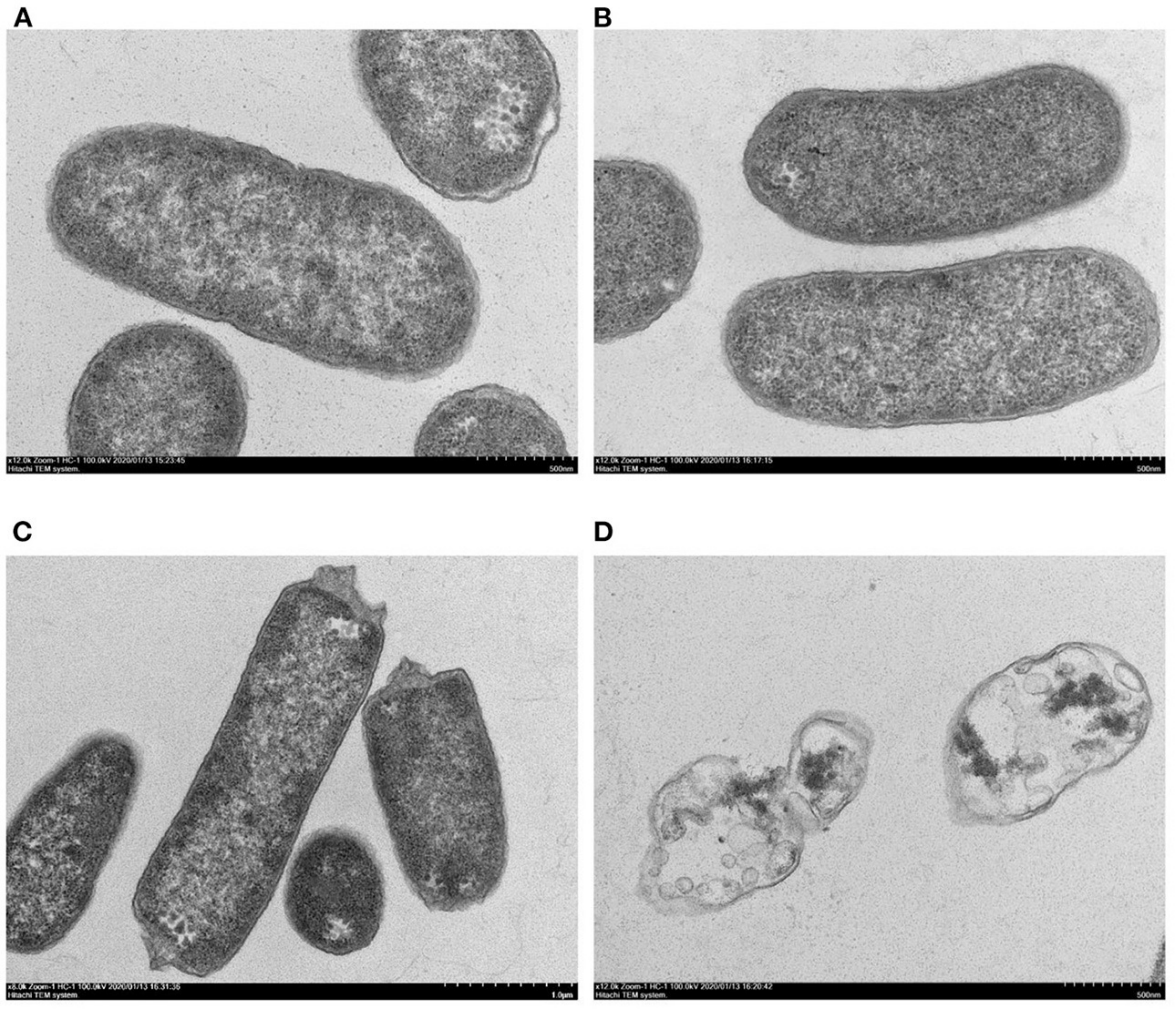
TEM of *E. coli* K12 and *E. coli* JW0688 with and without plantaricin BM-1. **(A,B)** show wild type *E. coli* K12 and *E. coli* JW0688 without plantaricin BM-1, and **(C,D)** show *E. coli* JW0688 and *E. coli* K12 with plantaricin BM-1.

Morphological changes in *E. coli* K12 and *E. coli* JW0688 with and without plantaricin BM-1 treatment were observed by SEM (Figure 3) and TEM (Figure 4). SEM showed that, in the absence of plantaricin BM-1, the bacterial morphology of *E. coli* K12 and *E. coli* JW0688 was not significantly different. The cells were short, rod-shaped, and smooth in appearance, and the edges were intact (Figures 3A,B). In the presence of plantaricin BM-1, the morphology of *E. coli* JW0688 was not obviously different than the morphology without plantaricin BM-1; the cell surface showed a slight fold contraction, the overall edge was intact, and there was no obvious rupture (Figure 3C). However, the morphology of *E. coli* K12 significantly changed in the presence of plantaricin BM-1; the cell surface was incomplete, the edges were uneven, and the surface of the cell showed obvious pitting and rupture (Figure 3D).

TEM was used to observe structural details within the bacterial cells (Figure 4). In the absence of plantaricin BM-1, the bacterial morphology of the *E. coli* K12 and *E. coli* JW0688 strains was not significantly different, and both bacteria showed uniform internal structures and smooth and complete appearance (Figures 4A,B). However, in the presence of plantaricin BM-1, the cytoplasm of *E. coli* JW0688 showed a slight contraction (Figure 4C). In contrast, the cytoplasm of *E. coli* K12 had contracted significantly in the presence of plantaricin BM-1, the cell surface had ruptured, the contents leaked out, and the outer membrane was separated from the inner membrane. Thus, the cell morphology was not maintained (Figure 4D).

### Proteomic Analysis

**Figure 5:**
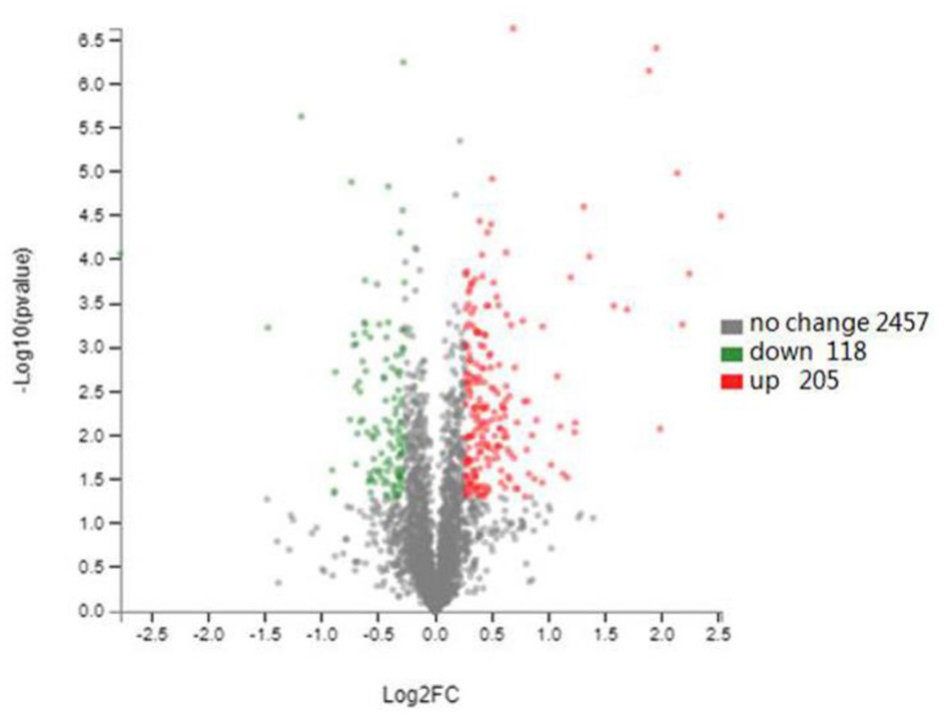
Changes in the *E. coli* K12 proteome after *ybfA* deletion. Volcano plots of 2780 identified proteins are shown. The colors indicate the fold changes. Red represents proteins with the fold changes greater than 1.20, and green represents proteins with the fold changes <0.83 (*P* < 0.05).

TMT labeling-based proteomics was applied to characterize the effect of plantaricin BM-1 on *E. coli*. A total of 2,780 proteins were identified, and 323 proteins were differentially expressed in *E. coli* JW0688 using a threshold of 1.2-fold (*P* < 0.05, Figure 5). Among the 323 differentially expressed proteins, 284 proteins were labeled as cell components (CC), and the content related to membrane proteins was 56.04%. Of these 323 differentially expressed proteins, 118 were downregulated and 205 were upregulated in *E. coli* JW0688 when compared with *E. coli* K12.

Among the downregulated proteins, many proteins related to biological pathways (BP) were significantly enriched, including localization (GO: 0051179), cellular component organization or biogenesis (GO: 0071840), biological regulation (GO: 0065007) and regulation of biological process (GO: 0050789). In terms of molecular function (MF), binding (GO: 0005488), catalytic activity (GO: 0003824) and transporter activity (GO: 0005215) were significantly enriched (Table 4). Among the downregulated differential proteins, the HTH-type transcriptional regulator HdfR (P0A8R9), which negatively regulates the transcription of the main flagellar operon, was downregulated 2.27 fold, and the expression of quorum-sensing molecule AI-2 in the receptor protein transcriptional regulator LsrR (P76141) was reduced 1.35 fold.

**Table 4:** GO categories for the down-regulated proteins in *E. coli* JW0688.

**Term type**	**GO term**	**GO ID**	**JWE0688 vs. K12 percent**
Molecular function	Binding	GO:0005488	83/118
Molecular function	Catalytic activity	GO:0003824	82/118
Molecular function	Transporter activity	GO:0005215	36/118
Molecular function	Nucleic acid binding transcription factor activity	GO:0001071	7/118
Biological process	Cellular component organization or biogenesis	GO:0071840	25/118
Biological process	Biological regulation	GO:0065007	24/118
Biological process	Regulation of biological process	GO:0050789	21/118
Biological process	Localization	GO:0051179	41/118
Cellular component	Organelle part	GO:0044422	5/118
Cellular component	Membrane-enclosed lumen	GO:0031974	4/118

Among the upregulated proteins, contains 117 membrane proteins (GO:0016020),these included 16 outer membrane proteins (GO: 0019867) and 15 cell outer membrane proteins (GO: 0009279); membrane part (GO: 0044425), cell part (GO: 0044464), and macromolecular complex (GO: 0032991) were significantly enriched in the cellular component. In addition, among the upregulated proteins, a variety of proteins related to biological pathways were significantly upregulated, including cellular process (GO: GO:0009987), metabolic process (GO: 0008152), single-organism process (GO:0044699), and response to stimulus (GO: 0050896) (Table 5). In biological pathways, we found that the expression of the BasS/BasR two-component system was significantly upregulated in *E. coli* JW0688.

**Table 5:** GO categories for the up-regulated proteins in *E. coli* JW0688.

**Term type**	**GO term**	**GO ID**	**JWE0688 vs. K12 percent**
Biological process	Cellular process	GO:0009987	180/205
Biological process	Single-organism process	GO:0044699	163/205
Biological process	Response to stimulus	GO:0050896	69/205
Biological process	Metabolic process	GO:0008152	162/205
Cellular component	Membrane	GO:0016020	117/205
Cellular component	Membrane part	GO:0044425	98/205
Cellular component	Cell part	GO:0044464	180/205
Molecular function	Molecular transducer activity	GO:0060089	15/205
Molecular function	Electron carrier activity	GO:0009055	12/205

### Mechanism by Which the BasS/BasR TCS Regulate the Sensitivity of *E. coli* to Bacteriocins

**Figure 6:**
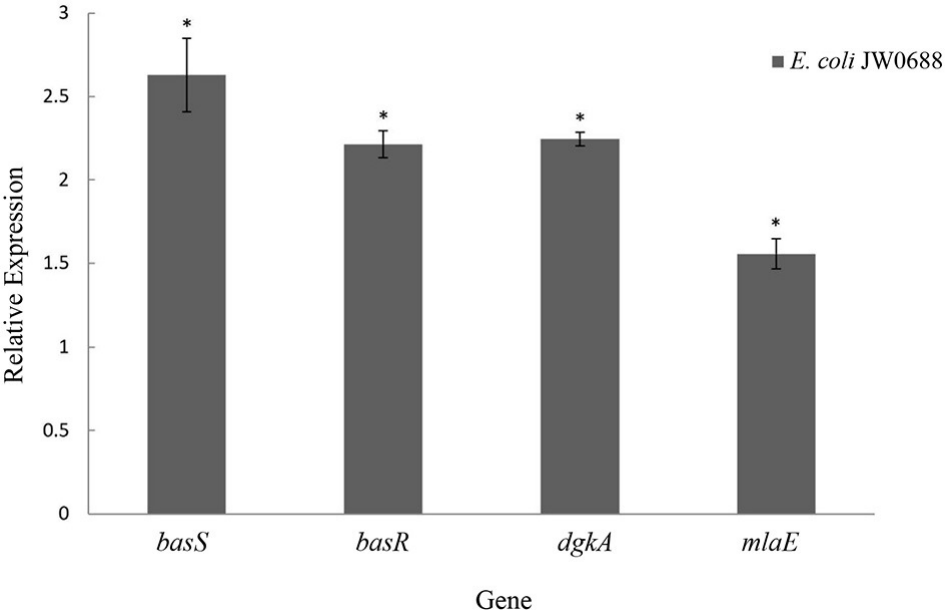
Expression levels of BasS/BasR two-component system and its downstream regulatory genes in *E. coli* JW0688. *Means significant result.

As show in Table 6, compared with wild-type *E. coli*, the expressions of BasR (P30843) and BasS (P30844) were respectively 2.24 and 2.26 times higher in *E. coli* JW0688. Proteins that are targets of BasR include MlaE (P64606), PutF (P07117), DgkA (P0ABN1), PotF (P31133), and others that also increased to a certain extent (Ogasawara et al., 2012). In addition, RT-qPCR results (Figure 6) also showed that the transcriptional levels of *basS, basR, mlaE*, and *dgkA* of *E. coli* JW0688 were all increased significantly (*p* < 0.05). These results also supported proteomic results, indicated that the expression of BasS/BasR TCS and its downstream regulatory genes in *E. coli* increased by *ybfA* gene mutation.

**Table 6:** BasS/BasR two-component system in *E. coli* JW0688 regulates protein expression changes.

**Accession number**	**Description**	**Fold change**	***P* value**	**Protein**	**Function**
P30843	Transcriptional regulatory protein BasR	2.24	0.000184	BasR	Transcription
P30844	Sensor protein BasS	2.26	0.0251	BasS	Autophosphorylation
P0ABN1	Diacylglycerol kinase Diacylglycerol kinase	1.28	0.03058	DgkA	Cell wall/membrane/envelope biogenesis
P64606	Intermembrane phospholipid transport system permease protein MlaE	1.23	0.01298	MlaE	Cell wall/membrane/envelope biogenesis
P23827	Ecotin	1.22	0.01075	eco	Unknown
P04949	Flagellin	4.73	0.000144	FliC	Cell motility
P77398	Bifunctional polymyxin resistance protein ArnA	2.15	0.00000206	ArnA	Cell wall/membrane/envelope biogenesis
P77690	UDP-4-amino-4-deoxy-L-arabinose–oxoglutarate aminotransferase	1.92	0.000148	ArnB	Cell wall/membrane/envelope biogenesis
P27127	Lipopolysaccharide 1,6-galactosyltransferase	0.76	0.002109	RfAB	Cell wall/membrane/envelope biogenesis
P07117	Sodium/proline symporter	2.18	0.000013	PutP	Cell wall/membrane/envelope biogenesis
P31133	Putrescine-binding periplasmic protein	1.94	0.0001931	PotF	Cell wall/membrane/envelope biogenesis

### Sensitivity Analysis of the BasS/BasR Two-Component System in *E. coli* to Plantaricin BM-1

**Figure 7:**
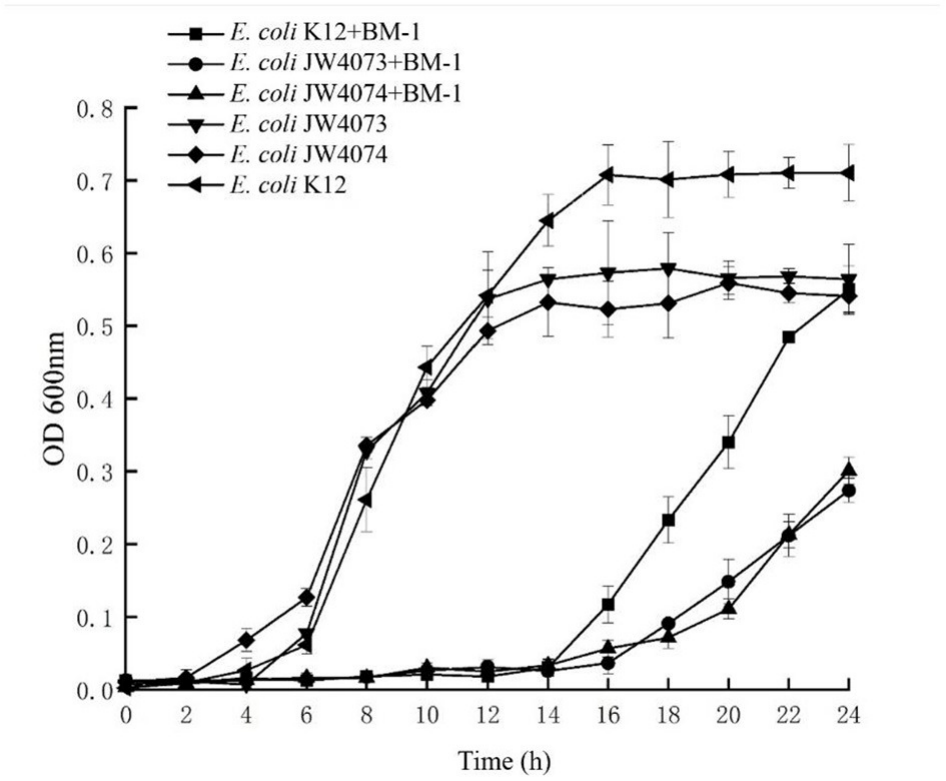
Effect of plantaricin BM-1 on the growth of wild type *E. coli* K12, *E. coli* JW4073, and *E. coli* JW4074. ■, • and ▴ represent the OD_600_ of wild type *E. coli* K12, *E. coli* JW4073, and *E. coli* JW4074 in the presence of plantaricin BM-1 (3× MIC), respectively. ◂, ▾ and ♦ represent the OD_600_ of wild type *E. coli* K12, *E. coli* JW4073, and *E. coli* JW4074 without the presence of plantaricin BM-1, respectively.

The MIC of *E. coli* JW4073 and *E. coli* JW4074 to plantaricin BM-1 were 0.46 and 0.61 mg/mL, all of which significantly decreased compared with that for wild type *E. coli* and Figure 7 shows that the growth curves of *E. coli* K12, *E. coli* JW4073, and *E. coli* JW4074 with and without the presence of plantaricin BM-1. During the experiment time, the three strains cultured without plantaricin BM-1 showed a typical growth trend. However, in the first 14 h, the three strains without the presence of plantaricin BM-1 showed no obvious growth. Therefore, growth was inhibited in the first 14 h. Beginning on the 16th hour, the growth of all three strains increased significantly. At 24 h, the OD_600_ of *E. coli* K12 was 0.550, while the OD_600_ of *E. coli* JW4073 and *E. coli* JW4074 were 0.274 and 0.301, respectively, (*p* < 0.05). Based on the OD_600_ values of *E. coli* K12, *E. coli* JW4073, and *E. coli* JW4074 at 24 h, compared with *E. coli* K12, *E. coli* JW4073 and *E. coli* JW4074 were more sensitive to plantaricin BM-1, that is, plantaricin BM-1 had a more obvious inhibitory effect on the *E. coli* strains that did not express the BasS/BasR two-component system.

## Discussion

IIa bacteriocins are small peptides that significantly inhibit the activity of the gram-positive bacterium *Listeria monocytogenes* (Hu et al., 2012). The antibacterial mechanism of IIa bacteriocins in gram-positive bacteria involves the mannose permeable enzyme ${\text{EII}}_{\text{t}}^{\text{Man}}$, which is located on the bacterial cell membrane, as the receptor. ${\text{EII}}_{\text{t}}^{\text{Man}}$ is part of the phosphotransferase system, which is involved the phosphorylation and transport of sugars in some bacteria (Stoll and Goebel, 2010). In *L. monocytogene*s, ${\text{EII}}_{\text{t}}^{\text{Man}}$ is composed of three subunits, IIA and IIB, which comprise the hydrophilic phosphotransferase domain located in the cytoplasm that is involved in phosphorylation and IIC and IID, which are hydrophilic phosphoryl transferase domains located on the membrane that are involved in mannitose transport (Kjos et al., 2011). In *E. coli*, the mannose phosphotransferase system has been shown not to be a bacteriocin receptor (Kjos et al., 2009), and *E. coli* can maintain the ultrastructure of cells by accelerating peptidoglycan synthesis and regulating the expression of membrane proteins to resist the damage of bacteriocins (Wang et al., 2020a,b).

The cell membrane of *E. coli* is the primary barrier by which defend against external harmful substances. Bacteriocins affect bacteria through adsorption and insertion into the cell membrane, resulting in increased membrane permeability, holes, leakage of cell contents, and finally collapse and death (Huang et al., 2010; Cui and Guo, 2018). The barrier effect of the cell membrane is important for resistance to antimicrobial peptides and can protect bacteria from external antimicrobial substances (Liu et al., 2013a,b). Thus, the ability of bacteria to maintain and modify their cell membranes is critical to resistance (Chen and Groisman, 2013).

In this study, an *E. coli* strain with an *ybfA* mutation (*E. coli* JW0688) was used. Studies have shown that YbfA is associated with sensitivity to radiation (Neil et al., 2016) and is a carrier gene of type 2 integrants (Wang et al., 2019). YbfA is also a predicted inner membrane protein. In our growth curve experiment, *E. coli* JW0688 showed reduced sensitivity to plantaricin BM-1, proteomic analysis and RT-qPCR showed that the expression of the BasS/BasR two-component system and its downstream genes were increased in the *ybfA* deletion strain. Previous studies have shown that the *E. coli* BasS/BasR two-component system can regulate the composition of outer membrane lipopolysaccharide or the expression of modification genes through targets, thereby controlling the formation and function of the membrane (Ogasawara et al., 2012) and enhancing membrane permeability and altering the resistance to antimicrobial peptides (Lee et al., 2005). The BasS/BasR two-component system is an iron-zinc induction transcription regulator that is mainly involved in the reaction to divalent metal ions (Fe, Zn) that while essential are also toxic to *E. coli* (Ogasawara et al., 2012). The regulatory mechanism is similar to that of the PmrA/PmrB two-component system of *Salmonella*. BasS protein, which is responsible for receiving environmental signals, is stimulated by external signals. The HATPase domain on BasS provides energy for the hisKA domain, phosphorylates the histidine in this domain, and sends a signal to BasR activating it. The BasR protein further regulates other target genes to respond to external stimuli (Gunn, 2008; Yu et al., 2015). Target genes that are activated by BasR usually contain a fixed BasR Box binding sequence consisting of five nucleosides. Studies have shown that some proteins in *E. coli* contain this binding sequence and have been proven to be BasR targets. These proteins are related to membrane structure and function (Ogasawara et al., 2012). The proteomics experiment showed that DgkA (P0ABN1), MlaE (P64606), PutF (P07117), and PotF (P31133) are downstream proteins regulated by BasR, and their expression levels were significantly changed. These proteins are mainly responsible for regulating cell membrane function and increasing the rigidity of the cell outer membrane (Ogasawara et al., 2012). The increased expression of these proteins indicates that excessive expression of the BasS/BasR two-component system in *E. coli* K12 can stabilize the expression of *E. coli* membrane proteins, maintain the function of the cell membrane, and promote the stability of the outer membrane (Ogasawara et al., 2012).

In summary, the *ybfA* gene in *E. coli* K12 regulates the expression of the BasS/BasR two-component system: when the *ybfA* gene is knocked down, the expression of BasS/BasR two-component system in *E. coli* K12 is increased, which directly affects the expression of proteins regulated by this two-component system. In this study, we found that the expression of the BasS/BasR two-component system was upregulated by *ybfA* gene deletion, which promoted cell membrane modifications in *E. coli*, which protected the bacteria and reduce the damage caused by the bacteriocin. In conclusion, YbfA regulates plantaricin BM-1 sensitivity in *E. coli via* the BasS/BasR two-component regulatory system.

## Data Availability Statement

The datasets presented in this study can be found in online repositories. The names of the repository/repositories and accession number(s) can be found below: iProX, IPX0002721000.

## Author Contributions

XC: conceived and designed the experiments, performed the experiments, and analyzed the data. YL: analyzed the data. JJ, HL, and YH: contributed materials. HZ: conceptualization and methodology. YX: conceived and designed the experiments. All authors contributed to the article and approved the submitted version.

## Conflict of Interest

The authors declare that the research was conducted in the absence of any commercial or financial relationships that could be construed as a potential conflict of interest.

## Publisher's Note

All claims expressed in this article are solely those of the authors and do not necessarily represent those of their affiliated organizations, or those of the publisher, the editors and the reviewers. Any product that may be evaluated in this article, or claim that may be made by its manufacturer, is not guaranteed or endorsed by the publisher.
